# Investigation of Coronavirus disease 2019 virus in vaginal fluid and menses blood and the effect on menstrual cycle duration and sexual desire: A cross-sectional study

**DOI:** 10.18502/ijrm.v21i8.14019

**Published:** 2023-09-20

**Authors:** Leila Sadeghi, Laya Farzadi

**Affiliations:** ^1^Department of Obstetrics and Gynecology, Alzahra Hospital, Tabriz University of Medical Sciences, Tabriz, Iran.; ^2^Women's Reproductive Health Research Center, Tabriz University of Medical Sciences, Tabriz, Iran.

**Keywords:** Coronavirus, COVID-19, Vagina, Menstruation.

## Abstract

**Background:**

Coronavirus disease 2019 (COVID-19) was detected in the throat, urine, and feces but has little evidence documented of sexual transmission.

**Objective:**

Here, we aimed to diagnose the presence of COVID-19 in vaginal fluids and menses blood. Menstrual cycle duration and sexual desire were the other aims.

**Materials and Methods:**

In this cross-sectional study, 300 individuals with clinical approval of COVID-19 infection who were referred to the Alzahra hospital of Tabriz University of Medical Sciences, Tabriz, Iran were divided into mild (n = 178, partial pressure of oxygen ≥ 91) and severe (n = 122, partial pressure of oxygen ≤ 91) groups, also based on clinical signs and hospitalization, from January to May 2021. Demographic characteristics, menstruation, and sexual desire of individuals were recorded in the questionnaire blood sampling was done on days 2-4 for menses, and vaginal fluid after menses for polymerase chain reaction by using a Dacron tip swab.

**Results:**

Participants were studied in the mild (mean age: 43.32 ± 7.41) and severe (mean age: 47.15 ± 6.9) groups. COVID-19 infection resulted in shortening the menstrual cycle duration in the severe group (30.15 ± 2.9 vs. 25.12 ± 2.1 days, p = 0.01). Polymerase chain reaction test for vaginal fluid and menses blood was negative for all cases. Sexual desire declined in both groups, significantly.

**Conclusion:**

This virus was not present in the menses blood and vaginal fluid of women with COVID-19 infection, which proposed a low risk of virus transmission via vaginal tracts. Severe COVID-19 infection may affect the menstrual duration.

## 1. Introduction

In late December 2019, an outbreak of the coronavirus disease 2019 (COVID-19) happened in China that rapidly spread all over the world. This phenomenon became a main disaster affecting the public health (1-3). COVID-19, known as severe acute respiratory syndrome coronavirus 2 (SARS-CoV-2) (4), caused more than 3 million deaths worldwide (5, 6). The key pathogenicity of COVID-19 was related to the human angiotensin-converting enzyme 2 receptors (ACE2) (7), which are expressed in various organs, especially in airways cells, reproductive systems, heart, intestine, kidneys, and placenta (8). The principal way of virus transmission was the droplets of respiratory secretions, including cough, sneezing, and close contact with an infected person (9).

However, few reports have documented the presence of COVID-19 in other parts, such as vaginal fluids, feces, urine, and blood (10). One of the concerns about COVID-19 was the risk of the virus spreading through the reproductive system, which could probably cause infertility and pregnancy complications (11). Also, the SARS-CoV-2 virus was identified in the testis of infected individuals (12). However, SARS-CoV-2 was not detected in the uterus of female individuals (13).

A previous study in 2020 reported negative tests for the COVID-19 virus in vaginal and cervical swabs of 42 individuals, except for one positive in anal swabs (14). Due to the small number of studies, further investigations were required to illuminate the possibility of virus transfection through sexual contact or vertical transmission from mother to child by investigating the presence of COVID-19 in vaginal secretion (15). ACE2 in ovarian granulosa cells of animals (16) raises the possibility that it might be a target for SARS-CoV-2 and consequently affect ovarian hormone production (1), resulting in menstrual cycle disorder.

The menstrual cycle is an essential physiological process affected by many biological, psychological, and social factors (17). Dysmenorrhea can negatively affect women's daily routines, job or educational performance, sleep quality, social life, and mental health (18). Since the beginning of the outbreak of COVID-19, there have been discussions on social media and blogs demonstrating that women have frequently experienced menstrual changes, such as menstrual duration, frequency, regularity, and volume, increased dysmenorrhea, and also worsened premenstrual syndrome (19).

Given the importance of COVID-19 transmission and the menstrual cycle for females, this study aimed to investigate the presence of COVID-19 in vaginal fluid and menses blood and investigate the effects of COVID-19 infection on menstrual cycle duration.

## 2. Materials and Methods

### Participants 

In this cross-sectional study, 300 individuals were enrolled with clinical approval of COVID-19 infection referring to Imam Reza and Sina hospitals of Tabriz, Iran, in January to May 2021. Participation was based on the following inclusion criteria, confirmed diagnosis of COVID-19 infection with reverse transcriptase-polymerase chain reaction (RT-PCR), an oxygen saturation level of less than 95% without supplementary oxygen. The exclusion criteria included, having endometriosis, vaginal infections, a history of ovarian dysfunction about 6 months prior to the onset of the disease, include a manifestation of delayed menses, menstrual cycle irregularities, or earlier menopause. Also, individuals unwilling to participate in the research were excluded.

### Data collection

Based on the partial pressure of oxygen (PO2), clinical symptoms, and hospitalization, individuals were divided into mild (n = 178, PO2 > 91) and severe (n = 122, PO2 < 91) groups. A questionnaire was documented, including information about demographic characteristics, such as age, body mass index, and underlying disease. All individuals were followed up for 3 months and asked to comment on the questionnaire items about their menstrual cycle duration (days) and sexual desire based on the frequency of sexual intercourse (per week) and respond to the items of the questionnaire before and after getting COVID-19 (20). Data were collected based on individuals self-report. The time span of 7 days considered as prolonged or shortened menstrual cycle in comparison to the average of the recent 3-month cycles in the same person before the COVID-19 infection.

### Detection of COVID-19 in vaginal fluid

In investigating the COVID-19 virus in the vaginal fluid, an RT-PCR assay was performed for 90 individuals who consented to the vaginal sampling. For this purpose, vaginal fluid was obtained by swab sampling during the COVID-19 infection according to the previously used method (13). For sampling we inserted a swab 2-3 cm into the women's vagina and then rotated. After that, swabs were transferred to the laboratory instantly, and a procedure by RT-PCR were performed using accepted protocols within 2 hr (21).

### Detection of COVID-19 in menses blood

For this purpose, sampling was done from hospitalized and outpatients whose nasopharyngeal RT-PCR was positive and showed symptomatic COVID-19 from the 2 ${}^{\text{nd}}$ -4 ${}^{\text{th}}$ day of menses. First, the speculum was placed in the vagina, followed by sampling with a swab from the menses blood in the cervical opening.

### Viral RNA isolation

A viral Nucleic Acid Extraction kit (Bioscience, Taipei, Taiwan, Cat. No. YVN50/YVN100) was used to extract the total viral RNA. In brief, the vaginal sample was transferred into a microcentrifuge tube, 400 µL veronal buffer and 10 µL proteinase K were added to the tube and incubated at 65 C for 10 min. Then, 500 µL of 95% ethanol was added to precipitate the RNA. Subsequently, the pellet was washed using washing buffer 1 and HIS-select (R) wash buffer after discarding the supernatant. Finally, the pellet was dispersed in 50 µL RNase-free water and saved for the PCR process.

### Real-time PCR 

The Novel Coronavirus Nucleic Acid Diagnostic Kit (PCR-fluorescence probing) of Sansure Biotech (Changsha, China) was used to amplify the nucleic acid of the COVID-19. In brief, 20 µL of the extracted RNA sample was mixed with 30 µL PCR-Mastermix and placed in the amplification equipment (Rotor-Gene 6000, Corbet Life Science, Australia). The Mastermix contained 2019-nCoV-PCR Mix, 2019-nCoV-PCR-Enzyme Mix, premiers, probes, dNTPs, MgCl2, RNasin, and PCR buffer for the 2019-nCoV-PCR Mix and RT enzyme and Taq enzyme for the 2019-nCoV-PCR-Enzyme Mix. The PCR program was regulated for 50 C (30 min) and 95 C (1 min) for reverse transcription and cDNA pre-denaturation. A 15-sec cycle at 95 C, followed by a 30-sec cycle at 60 C with 45 repetitions. Cycle threshold values of > 40 and < 40 were supposed to have negative and positive results, respectively.

### Ethical considerations

The study procedure was approved by the Ethical Committee of Tabriz University of Medical Sciences, Tabriz, Iran (Code: IR.TBZMED.REC.1399.816). On admission, participants provided written informed consent for using their medical records in research projects.

### Statistical analysis

Statistical analysis was conducted using SPSS version 17.0, Chicago, Illinois, USA (SPSS), and results were presented in percentage (%) or as means ± SD. Data were analyzed using paired sample *t* test and independent sample *t* test. Statistically significant differences were considered when p < 0.05.

## 3. Results

### Characteristics of the participants

A total of 300 individuals with confirmed COVID-19 were divided into mild and severe groups. There were 178 (59%) individuals in the mild group and 122 (41%) individuals in the severe group. The Mean of age in the mild and severe groups was 43.32 ± 7.41, and 47.15 ± 6.9, respectively. The mean oxygen saturation in the mild and severe groups was 92 ± 0.16 and 88 ± 0.34, respectively. The mean body mass index in the mild and severe groups was 26.4 ± 2.1 vs. 29.7 ± 2.6, respectively. The characteristics of the participants are shown in table I.

### COVID-19 virus detection in vaginal fluid 

Investigating COVID-19 in the vaginal fluid was evaluated in 90 individuals (37 individuals in the severe and 53 in the mild group). The cycle threshold value for all the samples was more than 40, which shows a lack of COVID-19 in vaginal fluid.

### COVID-19 virus detection in menses blood 

For this purpose, 30 menses blood samples were evaluated to detect the COVID-19 virus (15 individuals with severe and 15 with mild symptoms). Analysis did not show any COVID-19 infection in menstrual blood samples.

### Effects of COVID-19 infection on menstrual cycle duration 

**Figure 1:**
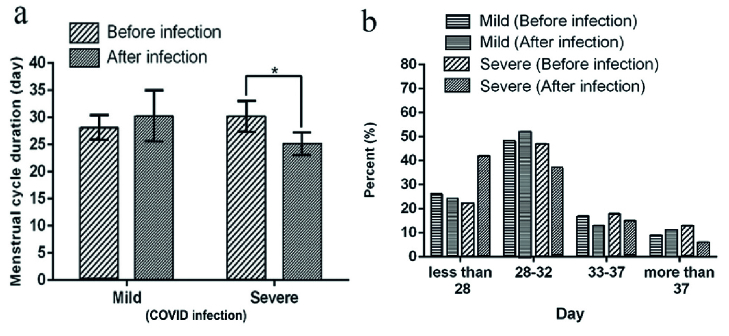
a) Comparison of menstrual cycle duration before and after the COVID-19 infection in mild and severe individuals. *Shows a significant difference (p < 0.05). b) The menstrual cycle distribution of individuals before and after COVID-19 infection in both mild and severe COVID-19 infection. The vertical graph shows the percentage of patients.

As shown in table II and figure 1a, in the mild group, the menstrual cycle duration of 3 months before and after the COVID-19 infection was 28.1 ± 2.3 (day) and 30.24 ± 4.7 (day), respectively, which shows no significant differences (p = 0.14). However, COVID-19 infection shortened the menstrual cycle duration in the severe group significantly (30.15 ± 2.9 days vs. 25.12 ± 2.1 days, p = 0.01). The menstrual cycle distribution is shown in figure 1b. After the COVID-19 infection, 42% of individuals with severe symptoms had a shorter menstrual cycle than the normal 28 days. However, 22% of these people had a shorter menstrual cycle than 28 days, even before the disease.

### Effects of COVID-19 infection on sexual desire

To compare the sexual desire of individuals before and after COVID-19, sexual intercourse frequency was recorded over a week. The results showed a decline in the libido after the COVID-19 infection compared to the pre-infection period in both mild and severe groups significantly (mild group: 2.6 ± 0.8 vs. 2.1 ± 0.5, p = 0.04; severe group: 2.9 ± 1.1 vs. 2.5 ± 0.8, p = 0.02) (Table II).

**Table 1:** Characteristics of the study participants (n = 300)


**Characteristics**		**Mild (n = 178)**	**Severe (n = 122)**	**P-value**
Age (yr)*		43.32 ± 7.41	47.15 ± 6.9	0.13
O_2_ saturation (%)*		92 ± 0.16	88 ± 0.34	< 0.001
BMI*		26.4 ± 2.1	29.7 ± 2.6	0.03
Alcohol use**		7 (3.9)	12 (9.8)	-
Smoking status**		10 (5.6)	7 (5.7)	-
History of menstrual disorders**		51 (28.7)	33 (27.1)	-
Place of residence**				
	**Urban**	107 (60.1)	90 (73.8)	-
	**Rural**	71 (39.9)	32 (26.2)	-
Disease (diabetes, hepatic disease, and malignant tumors)**				
	**Positive**	23 (12.9)	17 (13.9)	-
*Data presented as Mean ± SD. Independent *t* test. **Data presented as n (%). O_2_: Oxygen, BMI: Body mass index				

**Table 2:** Comparison of menstrual cycle and sexual behavior before and after COVID-19 infection


**Variables**		**3-5 months before the infection**	**3 months after the infection**	**P-value**
**Menstrual cycle duration (days)**				
	**Mild**	28.1 ± 2.3	30.24 ± 4.7	0.14
	**Severe**	30.15 ± 2.9	25.12 ± 2.1	0.01*
**Frequency of sexual intercourse (per week)**				
	**Mild**	2.6 ± 0.8	2.1 ± 0.5	0.04*
	**Severe**	2.9 ± 1.1	2.5 ± 0.8	0.02*
Data presented as Mean ± SD. Paired sample *t* test. *Shows a significant difference (p < 0.05)				

## 4. Discussion

Our study showed a negative result for COVID-19 in vaginal fluid samples. It has been suggested that it is less likely for the virus to transmit via vaginal tracts through sexual contact, normal delivery, or cesarean section. Analyzing the menstrual changes of individuals revealed that menstruation in individuals with severe infection manifests as shortened cycles. Moreover, laboratory analysis did not show any COVID-19 infection in menstrual blood samples. Also, both mild and severe infection groups experienced a lower libido during the illness.

Nowadays, COVID-19 has become one of the major health challenges worldwide (22). The invasion mechanism of the virus principally involves ACE2 in host cells and modulation of its expression. ACE2 is a pivotal component of the renin-angiotensin system, which could modulate angiotensin II (Ang II) and Ang-(1-7) levels. Recent evidence has suggested extensive expression of ACE2 in the ovary, vagina, and placenta. In addition, Ang II, ACE2, and Ang-(1-7) have a role in the regulation of the ovulation and development of follicles, luteal angiogenesis, and degeneration. Therefore, these findings are significant for an accurate understanding of the possibility of virus transfection either sexually, from mother to child, or during vaginal delivery (23).

Previous findings indicated that COVID-19 negative quantitative polymerase chain reaction results could not rule out the risk of infection and virus transmission through female genital systems. Using another virus detection method by TMA, they showed 20% positive results for COVID-19 in vaginal fluid samples, which were not detected by the RT-PCR test (24). It was reported that neonates delivered by COVID-19 pregnant mothers had no symptoms of infection. Furthermore, their study confirmed that COVID-19 positive conditions in pregnant women had no effects on postpartum hemorrhage, birth weight, and neonatal asphyxia rates (25).

One study investigated the different biological samples of women with COVID-19 at different stages of pregnancy, including vaginal secretions, stool, breast milk, neonatal throat, and anal swabs. They reported one positive stool and breast milk sample, but all vaginal fluids (n = 13), throat (n = 5), and anal swabs (n = 4) were negative for viral nucleic acid testing. So, negative results of COVID-19 testing in vaginal fluids proposed safe vaginal delivery (26).

The deficiency of reproductive-aged women with a small sample size restricted previous studies of SARS-CoV-2 detection in vaginal fluids. A research study examined 10 postmenopausal women with severe COVID-19 in the intensive care unit to detect SARS-CoV-2 in the vaginal fluid via RT-PCR assay. Test results did not detect a virus in vaginal fluids and suggested a bigger sample size for future studies (13).

Previous research detected SARS-CoV-2 in the vaginal fluid of postmenopausal and premenopausal individuals. In line with our results, previous study reported PCR-negative results for all vaginal fluid samples of 35 women with COVID-19. However, in another study the virus was not detected in the vaginal fluid of 28 pregnant women with COVID-19 (27). Also, in a study conducted on 13 samples of vaginal fluids, all individuals were negative for quantitative polymerase chain reaction (26). In another study conducted on 42 vaginal fluid samples and cervical swabs, all were negative for SARS-CoV-2, and one anal swab sample was positive. So, the authors demonstrated that the female lower genitalia might be a way for SARS-CoV-2 transmission (14).

Previous research monitored the menstrual cycle of 177 women with COVID-19 and showed that 45 (25%) individuals had menstrual volume changes, and 50 (28%) individuals had menstrual cycle changes, mainly a decline in the volume (20%) and a prolonged menstrual cycle (19%) (1). These results confirm the effects of COVID-19 infection on the ovaries, a regulator of menstruation. However, more investigations are needed to determine the exact impacts and molecular mechanisms involved in it.

## 5. Conclusion

This research confirms that the COVID-19 virus was not present in the menstrual blood and vaginal fluid of women with COVID-19 infection, which proposes a low risk of virus transmission via vaginal tracts. Furthermore, results confirm the menstrual duration change after severe COVID-19 infection, which shows that infection effects the ovaries. Moreover, COVID-19 infection significantly declines sexual intercourse and desire in both mild and severe COVID-19 infection groups. However, more investigations are needed to determine the exact impacts and molecular mechanisms.

##  Conflict of interest

The authors declare that there are no conflict of interest.
